# Constitutive secretion of pro-IL-18 allows keratinocytes to initiate inflammation during bacterial infection

**DOI:** 10.1371/journal.ppat.1011321

**Published:** 2023-04-17

**Authors:** Anders F. Johnson, Jenna S. Sands, Keya M. Trivedi, Raedeen Russell, Doris L. LaRock, Christopher N. LaRock

**Affiliations:** 1 Department of Microbiology and Immunology, Emory School of Medicine, Atlanta, Georgia, United States of America; 2 Department of Medicine, Division of Infectious Diseases, Emory School of Medicine, Atlanta, Georgia, United States of America; 3 Emory Antibiotic Resistance Center, Atlanta, Georgia, United States of America; Boston Children’s Hospital, UNITED STATES

## Abstract

Group A *Streptococcus* (GAS, *Streptococcus pyogenes*) is a professional human pathogen that commonly infects the skin. Keratinocytes are one of the first cells to contact GAS, and by inducing inflammation, they can initiate the earliest immune responses to pathogen invasion. Here, we characterized the proinflammatory cytokine repertoire produced by primary human keratinocytes and surrogate cell lines commonly used *in vitro*. Infection induces several cytokines and chemokines, but keratinocytes constitutively secrete IL-18 in a form that is inert (pro-IL-18) and lacks proinflammatory activity. Canonically, IL-18 activation and secretion are coupled through a single proteolytic event that is regulated intracellularly by the inflammasome protease caspase-1 in myeloid cells. The pool of extracellular pro-IL-18 generated by keratinocytes is poised to sense extracellular proteases. It is directly processed into a mature active form by SpeB, a secreted GAS protease that is a critical virulent factor during skin infection. This mechanism contributes to the proinflammatory response against GAS, resulting in T cell activation and the secretion of IFN-γ. Under these conditions, isolates of several other major bacterial pathogens and microbiota of the skin were found to not have significant IL-18-maturing ability. These results suggest keratinocyte-secreted IL-18 is a sentinel that sounds an early alarm that is highly sensitive to GAS, yet tolerant to non-invasive members of the microbiota.

## Author summary

Timely and appropriate inflammation is critical for an effective immune response against an invading pathogen. Some immune effectors are stored in an inert form that is enzymatically activated. This can allow for responses much more rapidly than transcriptionally regulation, on the order of minutes. An important regulator of these rapid responses is the inflammasome, which among its activities, processes the proinflammatory cytokines IL-1β and IL-18 into truncated forms that gain the ability to bind their cognate receptors. These cytokines are typically stored intracellularly until activation, but human keratinocytes constitutively secrete IL-18 in its unprocessed form. We show that this pool of IL-18 initiates an immune response to the human pathogen group A *Streptococcus*, but not non-pathogens. Mechanistically, this occurs from IL-18 cleavage by SpeB, a major virulence factor that is required for tissue invasion. Keratinocytes live in close association with diverse microbes and face a challenge in selectively responding to pathogens while tolerating normal microbiota. Our findings outline a strategy for discriminating species with high virulence potential through their use of proteolytic virulence factors and IL-18 as bait.

## Introduction

The skin provides the body’s outermost and first resistance to infectious, chemical, and physical insults. The obligate human pathogen Group A *Streptococcus* (GAS, *Streptococcus pyogenes*) colonizes oropharyngeal mucosa and epidermal surfaces, specifically adhering to and invading skin epithelial cells and keratinocytes [1–4]. Beyond superficial infections like impetigo, further tissue invasion can lead to cellulitis, sepsis, and necrotizing fasciitis. Invasive infections, and immune-mediated sequelae like rheumatic heart disease, are responsible for an estimated half a million annual deaths globally [5]. Inflammation typically serves to recruit and activate an antimicrobial immune response that is host-protective, but excess inflammation can contribute to tissue damage and complicates the treatment of invasive infections [6]. GAS induces inflammation by several mechanisms, including dedicated virulence factors like superantigens, and several lines of evidence suggest that at some body sites, inflammation can promote GAS replication and transmission [7–9]

Interleukin-18 (IL-18) is a proinflammatory cytokine that induces cell-mediated immunity [10]. IL-18 is detected by the IL-18R/IL-18RAP (IL18R1/IL1R7, IL-18Rα/IL-18Rβ) receptor complex and works with IL-12 to induce IFN-γ and Th1-type responses from T cells, NK cells, and dendritic cells [11–13]. Accordingly, IL-18 is important in the host defense against *Salmonella* Typhimurium [14], *Shigella flexneri* [15], *Yersinia enterocolitica* [16], *Listeria monocytogenes* [17], *Burkholderia pseudomallei* [18], *Mycobacterium tuberculosis* [19], *Streptococcus pneumoniae* [20], and group B *Streptococcus* [21]. Newly synthesized IL-18 (pro-IL-18) is inert and requires the removal of an amino-terminal autoinhibitory domain, which is canonically achieved by the host protease caspase-1 [11,12]. Caspase-1 is regulated by the inflammasome complex, which in myeloid cells can also regulate IL-1β maturation and the cell death program pyroptosis [22]. Caspase-8 [23], granzyme B [24], chymase [25], proteinase 3 [26], and neutrophil elastase [27] also cleave pro-IL-18, though their physiologic relevance in IL-18 activation remains to be established. Human keratinocytes constitutively express and release pro-IL-18 [28–32], suggesting the skin as an anatomical location where extracellular proteases participate in IL-18 activation.

We hypothesized that these early GAS interactions with human keratinocytes could contribute to the development of inflammation during infection. This study shows that keratinocytes release numerous proinflammatory cytokines during GAS infection, including pro-IL-18. During homeostasis, this form of IL-18 would not ordinarily be activated nor have proinflammatory activity. However, upon infection by GAS it is directly matured by the bacterial secreted cysteine protease SpeB, activating a proinflammatory cytokine cascade. This mechanism supports a model wherein SpeB acts as a “bacterial caspase” that proteolytically activates proinflammatory cytokines of the IL-1 family, which in humans may act as an early sentinel to limit GAS invasive infection and may broadly be a form of effector-triggered immunity against proteases.

## Results

### GAS induces keratinocytes secretion of proinflammatory cytokines

**Fig 1:**
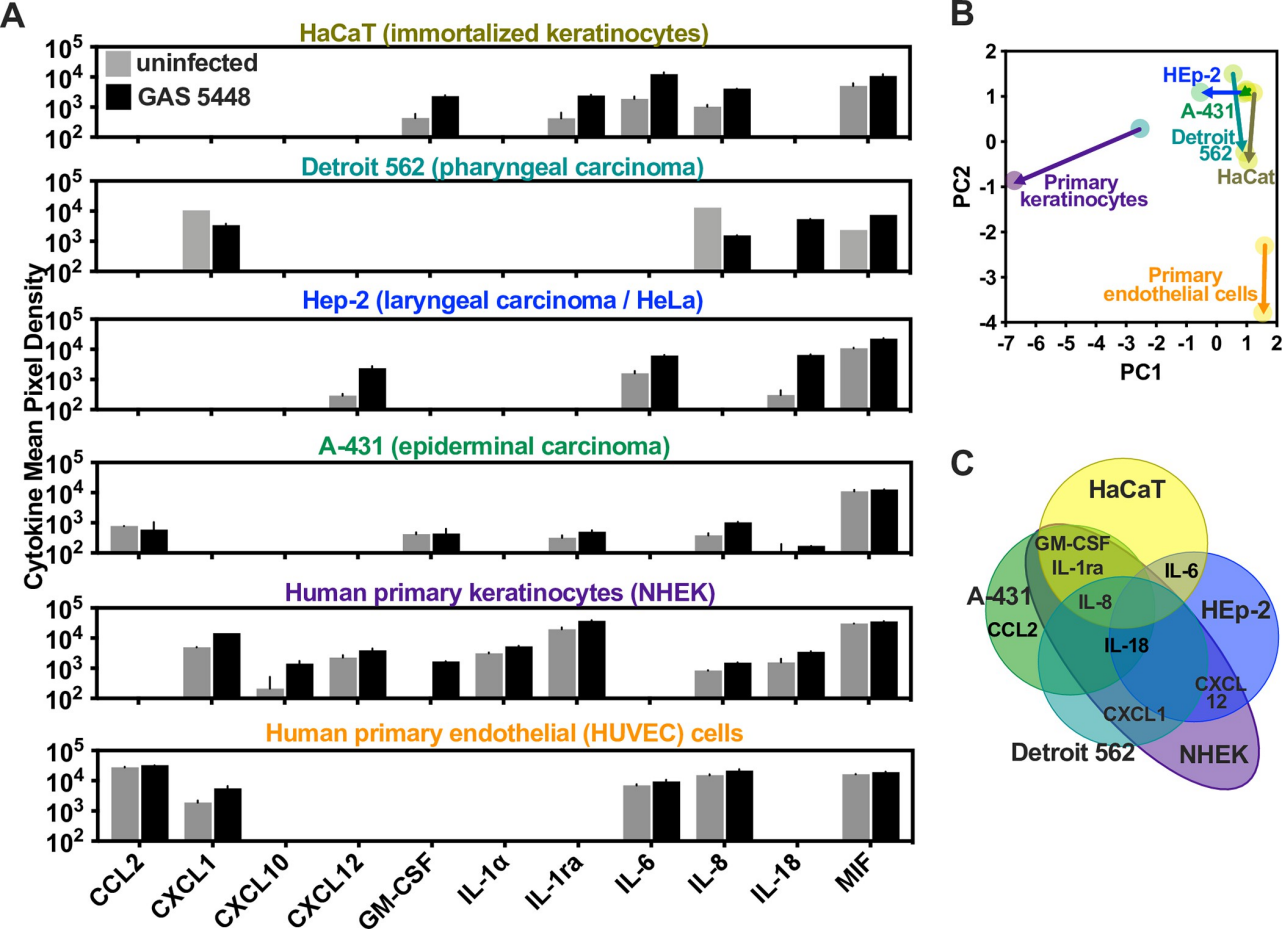
Cytokine profiles of keratinocytes and related cell lines. HaCaT, Detroit 562, HEp-2, A-431, primary keratinocytes, or HUVEC cells, were infected with 7.5 x 10^6^ colony-forming units (CFU) of GAS for 6 h. (A) Relative abundance of select cytokines was examined by membrane-based antibody array. (B) Cytokine profiles of each cell were examined by multivariate (principal component analysis), of the total variance, PC1 explains 48.67% and PC2 20.87%, from the raw cytokine quantities tabulated in (A). Arrows indicate change in cells from uninfected to 6 h infection. (C) Graphical representation of the congruent cytokine profiles between cell types.

The epithelium is one of the first body tissues GAS will contact. Therefore, resident cells provide one of the earliest responses to infection by producing antimicrobial effectors, proinflammatory cytokines, and chemokines that are necessary to effectively coordinate the immune response [33–36]. To assess the cytokine repertoire elicited by GAS, we infected immortalized and primary epithelial cells with M1T1 GAS 5448, a highly virulent strain associated with modern epidemic invasive infections [37]. Similar to previous observations [33,34,38], we observed robust secretion of macrophage migration inhibitory factor (MIF), IL-8 (CXCL8), and other proinflammatory cytokines and chemokines by infected HaCaT keratinocytes (**Fig 1A**). Detroit 562 human pharyngeal epithelial cells and A-431 human keratinocytes all secreted a similar, nonredundant cytokine profile. Hep-2 cells are one of the lines reported by the International Cell Line Authentication Committee (ICLAC) to contain characteristic HeLa cell markers and were apparently derived by HeLa contamination of human laryngeal adenocarcinoma cells [39]. We include it for context since extensive prior work has used it to model epithelial cell infection and found that it produces a cytokine repertoire partially overlapping other cell lines, as well as CCL2 (MCP-1), which is not characteristic of epithelial cells (**Fig 1A**).

Compared to each cell line, human primary keratinocytes (NHEK) expressed a greater diversity in CXC-family chemokines than any individual cell line and were the only ones that released IL-1α, a surface-tethered pro-inflammatory cytokine constitutively produced by healthy human epithelial cells (**Fig 1A**). Human primary endothelial cells (HUVEC) secreted a different repertoire of cytokines and chemokines, showing a distinct response (**Fig 1A**). No cell tested produced detectable CCL1, CCL5, G-CSF, IFN-γ, IL-1β, IL-2, IL-4, IL-5, IL-10, IL-12, IL-13, IL-16, IL-17, IL-17E, IL-21, IL-27, IL-32α, MIP-1, or TNF-α at a resting state or during infection with GAS (**S1 Fig**). Overall, primary cells formed distinct groups by multivariate analysis relative to all cell lines (**Fig 1B**) due to their distinct and expanded cytokine secretion profiles (**Fig 1C**) that diverged further during infection. Altogether, these results suggest that common cell lines do not fully recapitulate the cytokine response as it may happen *in vivo*, and caution should be used when modeling the inflammatory output of the host-pathogen interaction while using them.

### GAS activates IL-18

**Fig 2:**
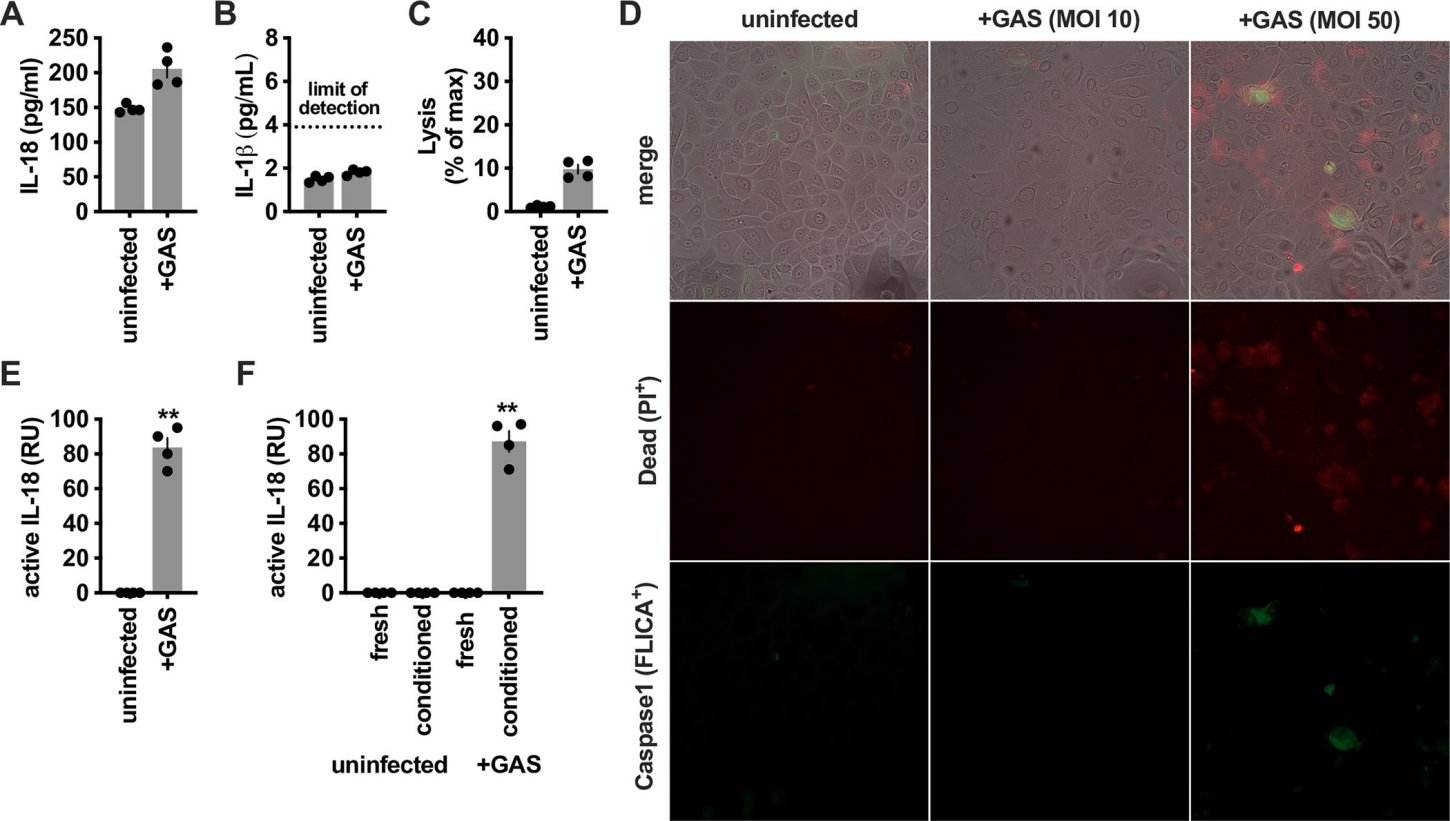
Examination of IL-18 activation in GAS-infected keratinocytes. Primary human keratinocytes were infected with GAS at a multiplicity of infection (MOI) of 10 for 4 h and (A) secreted IL-18 (total, pro- and mature forms) was measured by ELISA, (B) cell lysis measured by LDH release, (C) total IL-1β measured by ELISA, or (D) cells visualized by immunofluorescent microscopy with staining for cell death by propidium iodide uptake (red) and caspase-1 activation (green). (E) Secreted bioactive IL-18 measured with HEK-Blue IL18 reporter cells during infection as in (A). (F) Fresh or conditioned media were removed from primary human keratinocytes and incubated with GAS, then IL-18 activity was measured as in (E). Data were analyzed by 1-way ANOVA using Dunnett multiple comparisons analysis. All data represent at least 3 independent experiments with 4 replicates. Bars show median values ± standard deviation. **P <0.005; ns, not significant.

IL-18, a cytokine of the IL-1 family that bridges the innate and adaptive immune responses by stimulating IFN-γ production from T lymphocytes and NK cells [10], stood out for its expression pattern. In immune cells, IL-18 maturation is conventionally regulated by the inflammasome protease caspase-1, which also regulates its release through the cell death program, pyroptosis [40]. Thus, we expected to observe release only during infection, not from resting cells. In contrast, prior work has observed that primary human keratinocytes express a high level of pro-IL-18 and release it in their resting state without infection or further stimulation [29,41]. Consistent with these prior studies and **Fig 1A**, we observed that GAS infection was not required for IL-18 secretion, and there was significant release by healthy, uninfected keratinocytes (**Fig 2A**). IL-18 release is typically thought to require cell death, and we recently have shown that GAS induces cell death in keratinocytes via activation of the cell death effector Gasdermin A [42]. However, this IL-18 release did not require cell lysis (**Fig 2B**). Furthermore, no significant release of the inflammasome-regulated cytokine IL-1β was observed in the absence of infection, consistent with inflammasome-independent secretion of pro-IL-18 (**Fig 2C**). By microscopy, no cell lysis nor activation of inflammasome caspase-1 was observed under these infection conditions, though was increased at a higher multiplicity of infection (MOI) (**Fig 2D**).

We next examined whether the IL-18 secreted by keratinocytes was active. For cytokines that are post-translationally regulated by proteolysis, as for IL-18, activation is not necessarily specific to a single protease or cleavage site. Instead, there is potential for plasticity in this process, and the determinant of activity is exclusively whether the cleavage product(s) can bind and stimulate signaling through the cognate receptor [43]. To measure whether keratinocyte-released IL-18 had biological activity, we used HEK-Blue IL-18 Reporter cells, which have been engineered to express the IL-18 receptor complex (IL-18R and IL-18RAP) and induce an alkaline phosphatase reporter in response to IL-18, but not other cytokines or pathogen-associated molecular patterns. None of the IL-18 released by uninfected keratinocytes had activity; however, it was rendered active during GAS infection (**Fig 2E**). This suggested a GAS-dependent mechanism for the activation of IL-18. To exclude the possibility of the inflammasome or other keratinocyte factors in this activation, we removed supernatants from keratinocytes and incubated them with GAS. GAS was able to stimulate IL-18R signaling from this cell-free conditioned media (**Fig 2F**), suggesting conventional cellular regulators (the inflammasome) were not essential. Since no activation was seen by GAS alone, it does not directly stimulate IL-18R signaling, but instead, one or more GAS factors may directly act on the inert pro-IL-18 secreted by keratinocytes to convert it to an active form.

### GAS protease SpeB is required for the activation of keratinocyte IL-18

**Fig 3:**
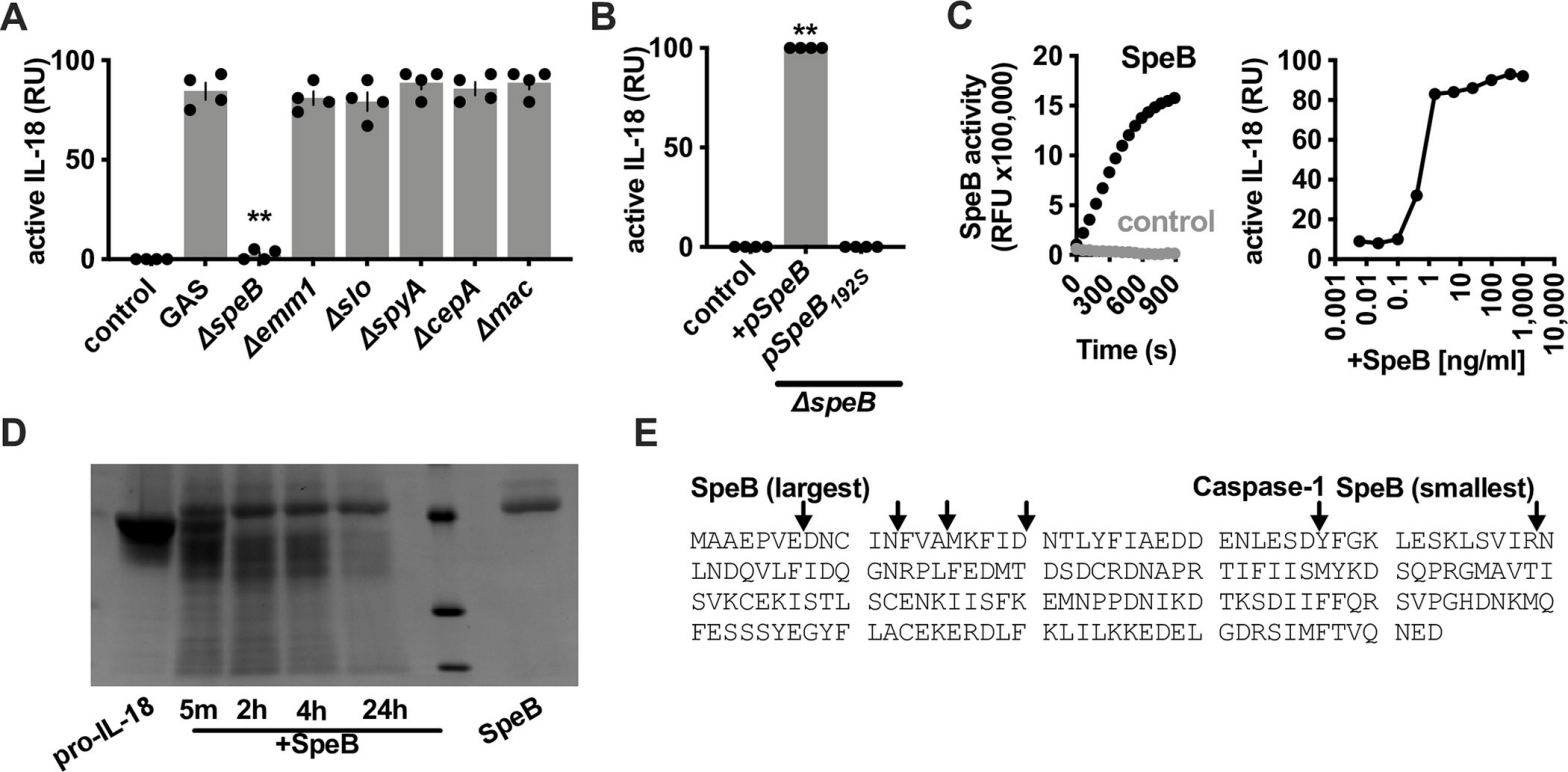
Examination of GAS requirements for IL-18 activation. Primary human keratinocytes were infected with GAS at MOI 10 for 4 h, then (A, B) bioactive IL-18 was measured with HEK-Blue IL18 reporter cells. (C) SpeB activity was measured using specific substrate sub103 and IL-18 activity measured in the supernatants from keratinocytes treated with titrations of purified SpeB protein. (D) Recombinant human pro-IL-18 was purified and incubated with purified, active SpeB, then cleavage products were separated by SDS-PAGE and visualized by staining. (E) Coding sequence of human IL-18 with probable and known cleavage sites indicated; the largest and smallest confirmed by Edman sequencing. Data were analyzed by 1-way ANOVA using Dunnett multiple comparisons analysis. All data represent at least 3 independent experiments with 4 replicates. Bars show median values ± standard deviation. **P <0.005; ns, not significant.

To determine the specific bacterial factors responsible for the direct maturation of pro-IL-18 by GAS, we screened a panel of defined virulence factor mutants. During keratinocyte infection, we found that the secreted cysteine protease SpeB was essential for generating active IL-18 (**Fig 3A**). Complementation of Δ*speB* GAS 5448 restored activation, while catalytically inactive SpeB_C192S_ did not, indicating the enzymatic activity of SpeB was required for IL-18 conversion (**Fig 3B**). Furthermore, in the absence of infection or other treatment, active SpeB alone was sufficient to activate IL-18 in a dose-dependent manner (**Fig 3C**). Taken together, SpeB is the GAS virulence factor responsible for IL-18 activation.

Conventional IL18 activation occurs by Caspase-1 removing an N-terminal pro-domain [40], freeing an ~18 kDa active C-terminal product to bind and form a signaling complex with IL-18R and IL-18RAP [44]. In contrast, SpeB initially truncated recombinant pro-IL-18 to 22 kDa which was further truncated down to several products ranging down to ~17 kDa (**Fig 3D**). SpeB will cleave after a Glu, Thr, Ala, Gly, Ser, Asn, or Asp that is preceded by a hydrophobic amino acid (Ile, Val, Phe, Tyr, or Met), particularly in regions rich in negatively-charged amino acids (Asp or Glu) [45,46]. The N-terminus of pro-IL-18 contains several potential cleavage sites for, matching these observations by SDS-PAGE and N-terminal cleavages confirmed Edman sequencing (**Fig 3E**).

### T cell activation by keratinocyte IL-18 requires SpeB

**Fig 4:**
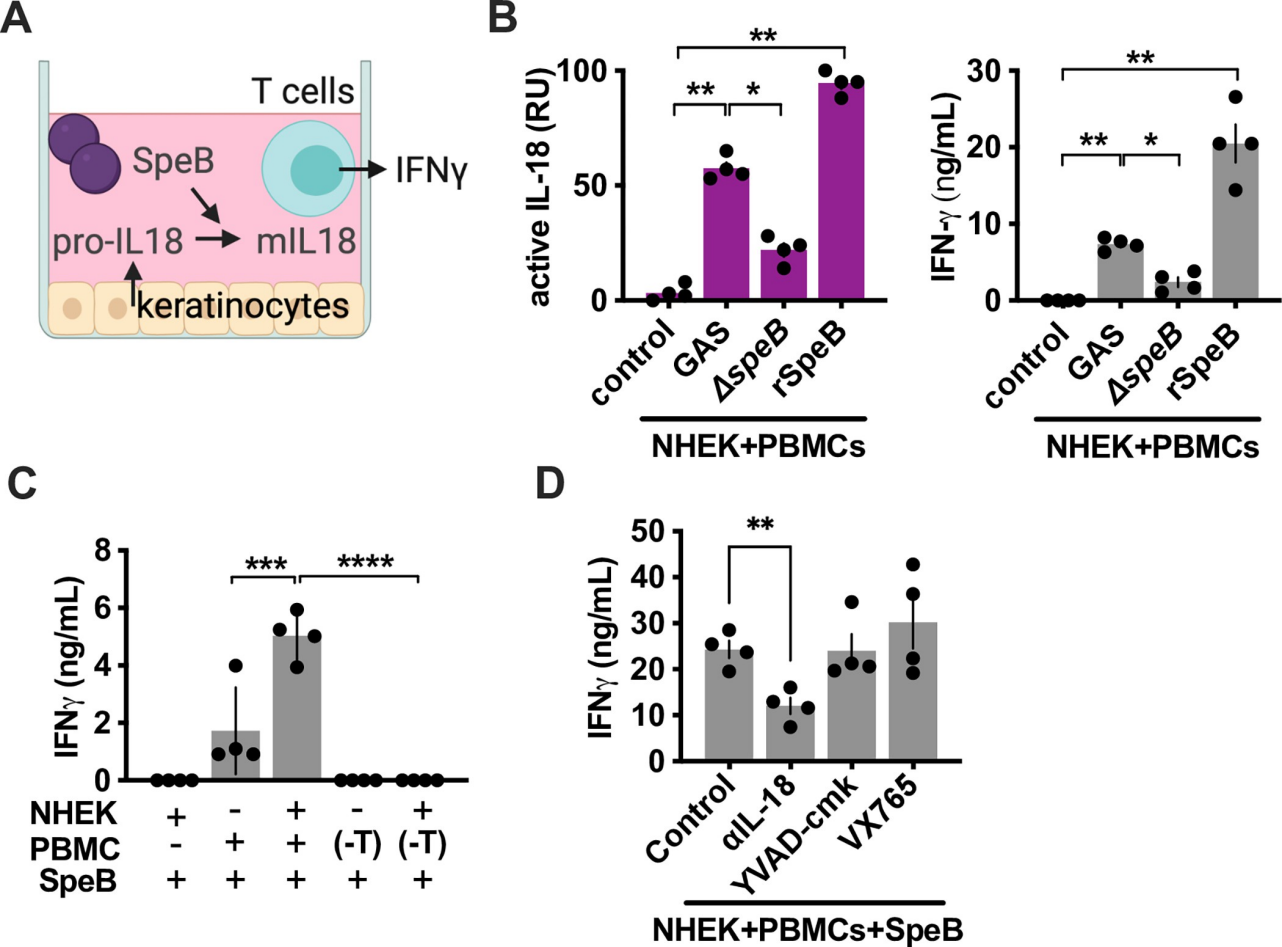
SpeB activation of IL-18 promotes antimicrobial IFN-γ responses. (A) Diagram of primary keratinocyte/PBMC co-culture model; IFN-γ is a reporter of T cell activation, which can occur via IL-18 and additional mechanisms during infection. (B) Cocultured keratinocyte/PBMCs were infected with 10^5^ CFU of GAS or treated with rSpeB. After 4 h, active IL-18 was quantified with HEK-Blue IL-18 reporter cells and IFN-γ by ELISA. (C, D) Cocultured cells were treated with SpeB as in (B), in combination or post-depletion of CD2^+^ T cells, or with treatments with IL-18 neutralizing antibody or 5 μM YVAD-cmk or 10 μM VX765 to inhibit inflammasome function. Data represent at least 3 independent experiments with 4 replicates. Data were analyzed by 1-way ANOVA using Dunnett multiple comparisons analysis. Bars show median values ± standard deviation. *P <0.05; **P <0.005; ns, not significant.

IL-18 was initially discovered for its ability to induce type II interferon (IFN-γ) from T cells [47]. Thus, we further examined whether the keratinocyte IL-18 processed by GAS is able to signal to induce IFN-γ production from human peripheral blood mononuclear cells (PBMCs) (**Fig 4A**). Keratinocytes alone did not induce IFN-γ from PBMCs, consistent with an insufficiency of the pro-IL-18 they release to signal or be activated by PBMCs (**Fig 4B**). However, during infection with SpeB-expressing GAS, there was activation of IL-18 signaling and production of and a proportionate SpeB-dependent increase in the production of IFN-γ (**Fig 4B**). Purified SpeB was sufficient to induce IFN-γ (**Fig 4B**), which required both NHEKs and specifically T cells and/or NK cells, since IFN-γ signaling was lost when CD2^+^ cells were depleted (**Fig 4C**). Consistent with an inflammasome-independent origin of this IL-18 signaling, IFN-γ was significantly decreased by anti-IL-18 neutralizing antibody, but not the inflammasome inhibitor drugs YVAD-cmk or VX765 (**Fig 4D**).

### IL-18 secretion is species-restricted

**Fig 5:**
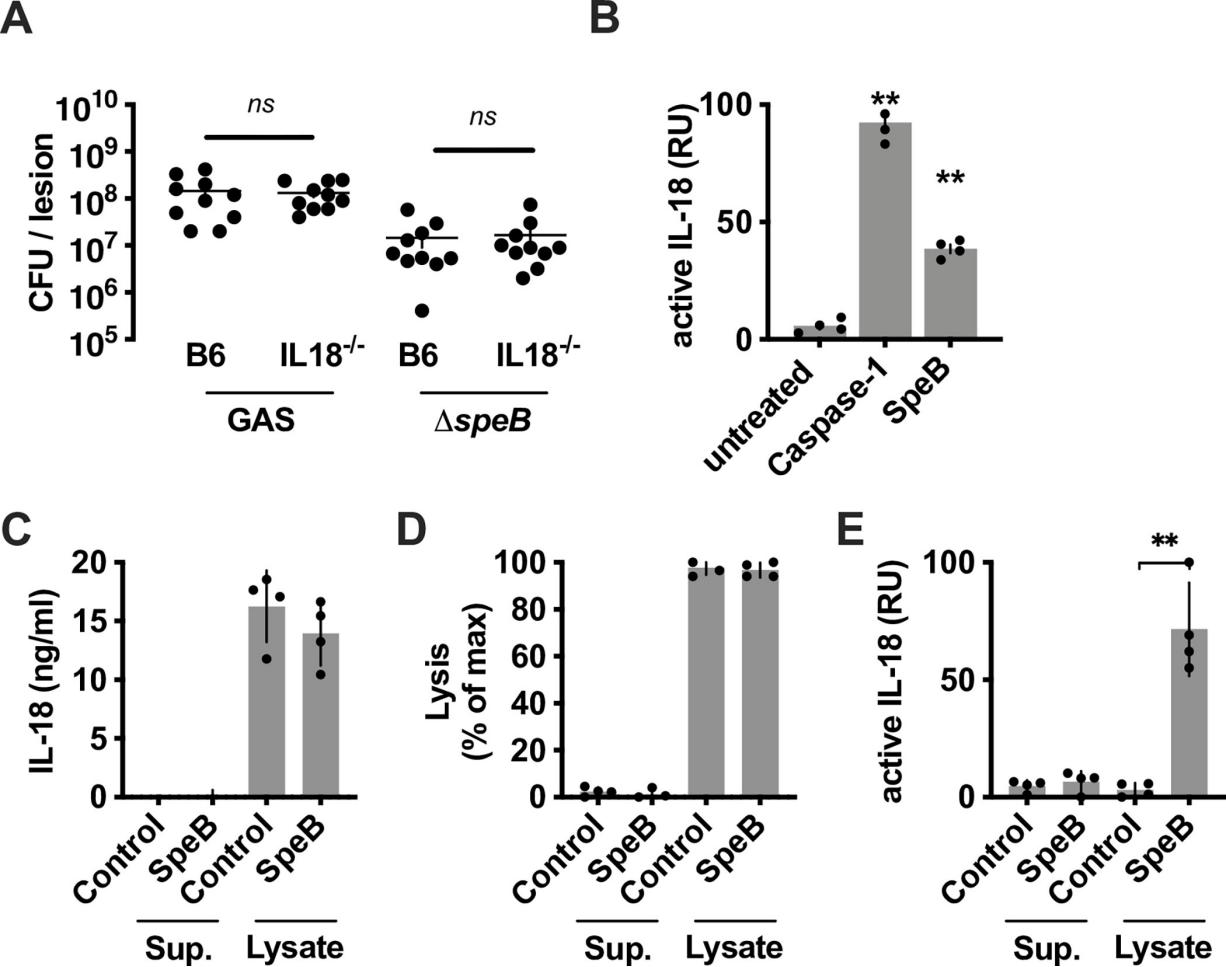
Mouse IL-18 can be activated by SpeB but is not secreted under normal inert conditions. (A) C57BL/6 wild-type or IL-18-knockout (*il18*^-/-^) mice were inoculated intradermally with 10^8^ CFU of GAS 5448 or its D*speB* mutant. After 72 h, mice were euthanized, and GAS CFU was enumerated at the infection site. Results are from 2 independent experiments with 5 mice in each. (B) Recombinant mouse pro-IL-18 was incubated with human Caspase-1 or SpeB and activation measured with HEK-Blue IL18 reporter cells. (C) Supernatants or lysates from mouse primary keratinocytes were examined for IL-18 by ELISA and (D) cell lysis confirmed by LDH release assay, or (E) incubated 4 h with SpeB, and active IL-18 was quantified with HEK-Blue IL-18 reporter cells. Data represent at least 3 independent experiments with 4 replicates. Data were analyzed by 1-way ANOVA using Dunnett multiple comparisons analysis. Bars show median values ± standard deviation. *P <0.05; **P <0.005; ns, not significant.

IL-18 and IFN-γ are critical in human immunity and broadly protective against infection [48–50], therefore, they are commonly studied using primary human cells such as whole blood or PBMCs [6,51–54]. Mice are frequently used to model infection, but only a few instances have *il18*^-/-^ mice are susceptible to infection [19,55–57]. This suggests that there could be differences in immune signaling between species. However, since IL-18 expression is elevated in experimental murine infections [43,52,58], we decided to examine whether IL-18 activation by SpeB could provide protective benefit in the typical skin infection model. No difference in GAS growth was observed between wild-type C57Bl/6 and *il18*^-/-^ mice (**Fig 5A**), suggesting no protective benefit in a model of acute infection. Since mice are not a natural host for GAS, we next sought to determine whether this was a species-dependent difference. Mouse pro-IL-18 has only 64.2% identity to human, with the greatest divergence in the N-terminal pro-domain. Nonetheless, SpeB-processed mouse pro-IL-18 to a biologically active product, albeit to a lesser extent than Caspase-1 (**Fig 5B**). However, when we examined primary mouse keratinocytes we found that, unlike human cells, they did not secrete detectable IL-18 into the supernatant (**Fig 5C**), but they did contain intracellular stores that could be released by chemical lysis (**Fig 5D**). This intracellular IL-18 lacked activity but could be converted to the active form when incubated with SpeB (**Fig 5E**). Thus, SpeB-dependent activation of keratinocyte IL-18 in mice is limited by differences in IL-18 secretion between species.

### IL-18 activation is microbial species-restricted

**Fig 6:**
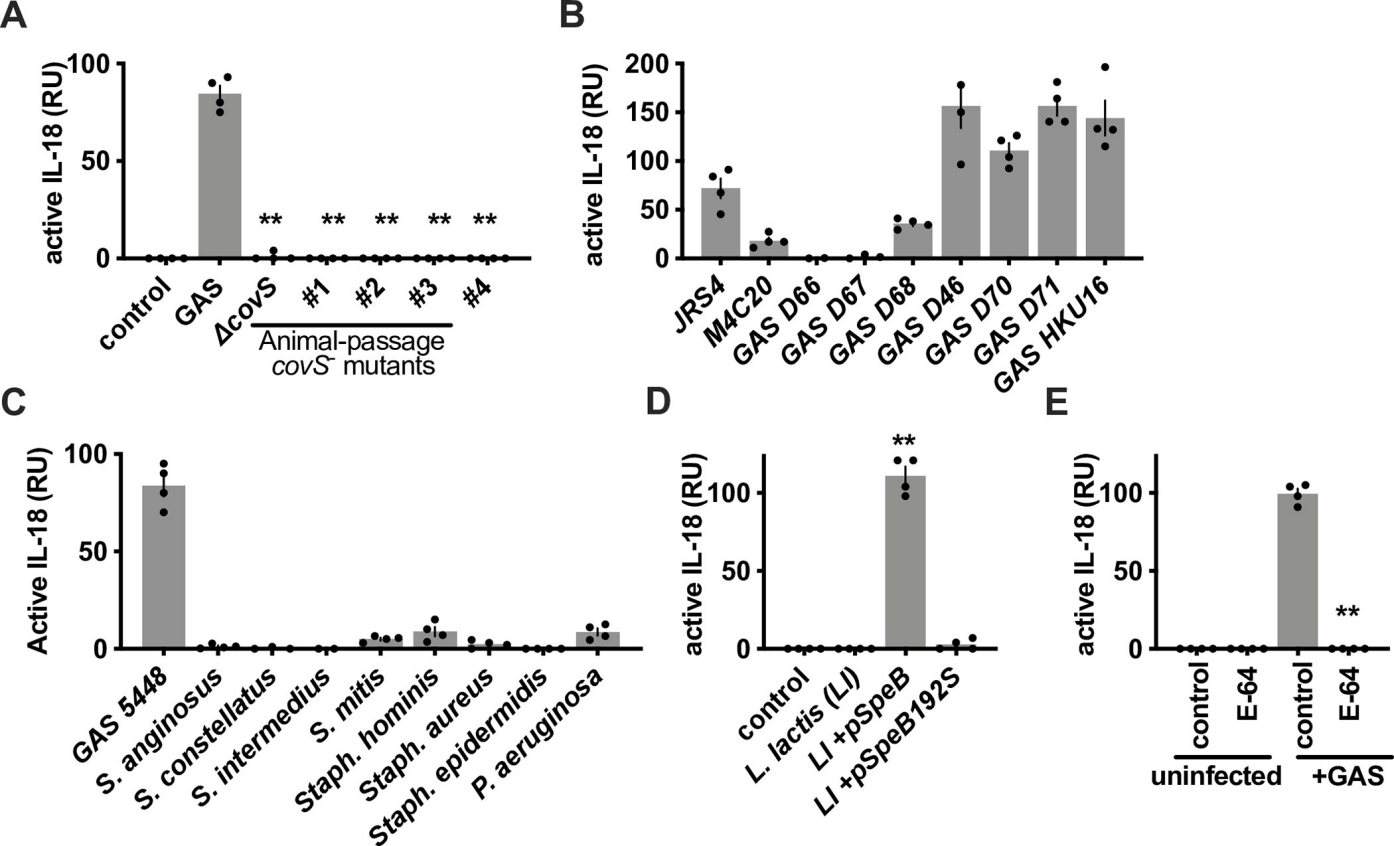
Bacterial activation of IL-18. (A, B) IL-18 activation was measured in the supernatants from keratinocytes infected with the indicated gene knockouts of GAS strain 5448 or wild-type GAS, and after 4 h, bioactive IL-18 was measured with HEK-Blue IL18 reporter cells. (C) IL-18 activation was measured in the supernatants from keratinocytes infected with the indicated bacterial species and after 4 h, bioactive IL-18 was measured with HEK-Blue IL18 reporter cells. (D, E) IL-18 activation was measured in the supernatants from keratinocytes infected with the indicated gene knockouts of GAS strain 5448, *L*. *lactis*, or during treatment with 5 μM E-64. Spectinomycin and anhydrotetracycline to maintain SpeB expression from the indicated plasmids. Data represent at least 3 independent experiments with 4 replicates. Data were analyzed by 1-way ANOVA using Dunnett multiple comparisons analysis compared to uninfected/untreated keratinocytes. Bars show median values ± standard deviation. *P <0.05; **P <0.005; ns, not significant.

GAS with mutations in the CovS component of the CovRS (CsrRS) two-component regulator naturally arise in human invasive infections and in murine models [59–61]. *covS* mutants no longer express SpeB, enter keratinocytes, or activate IL-1β or GSDMA [42,43]. Similarly, *covS* mutants do not activate keratinocyte-secreted pro-IL-18 (**Fig 6A**), and variable expression between clinical isolates leads to heterogeneity in whether any single clone activates IL-18 (**Fig 6B**). Many bacteria other than GAS secrete proteases, they are one of the most common protein classes and are frequently employed by pathogens as virulence factors. IL-1β, for example, is directly activated not only by SpeB, but also by *Pseudomonas aeruginosa* LasB and *Staphylococcus epidermidis* Esp [43,62,63]. These proteases potentially represent a group of “bacterial caspases” that may share the activation of these two related IL-1 family cytokines. Proteases such as these are often cell-density regulated and accumulate in overnight cultures of bacteria. Of these species and other major skin bacteria, under these conditions only GAS released proteases that activated the inert pool of pro-IL-18 secreted by keratinocytes (**Fig 6C)**. This suggests that constitutive pro-IL-18 secretion by human keratinocytes does not result in chronic inflammation, in part, because few proteases extracellularly convert IL-18 into an active form. In support of this, we tested gain-of-function using *Lactococcus lactis* made to express SpeB and found that the expression of catalytically active SpeB was sufficient for *L*. *lactis* to activate IL-18 (**Fig 6D**). Suggesting that targeting bacterial virulence factors can impact this process, treatment with E-64, a cysteine protease inhibitor active against SpeB, repressed GAS activation of IL-18 (**Fig 6E**).

## Discussion

SpeB is essential for GAS colonization throughout the body and penetrating deeper into tissue during invasive infection [43,64]. Skin keratinocytes are thus poised to mount some of the earliest immune responses against this important pathogen. Still, they must safeguard against aberrant activation by the microbiota since they are in constant contact with numerous other species they must tolerate. Consistent with prior observations, we observed constitutive pro-IL-18 secretion by primary human keratinocytes [28–31], (**Figs 1A** and **2A**) and discovered that it could be directly activated by SpeB (**Fig 3D**). Other bacterial species relevant for colonizing the skin do not similarly activate immature pro-IL-18 (**Fig 6C**), so we hypothesize that this pool of cytokine is poised for activation to serve as an early sentinel for infection by potentially invasive pathogens that make use of proteolytic virulence factors. By directly sensing specific proteases required for infection, IL-18 allows keratinocytes to discriminate between numerous species with high virulence potential and common pathogen-associated molecular patterns and toxins. Furthermore, there may be pathogens from genera other than *Streptococcus* with this activity since secreted proteases are broadly important for human pathogens and microbiota alike [65].

Diverse proteases generate a variety of IL-18 truncations, but how this impacts signaling activity has been unclear [24–27]. Recent NMR studies show that mature (caspase-1-cleaved) IL-18 has distinct spectra from pro-IL-18, suggesting that intramolecular interactions inhibit receptor binding from the pro-domain inducing significant structural changes in the cytokine [44]. Amino-terminal truncations showed removal of the first eight amino acids gave folding similar to pro-IL-18, the first ten an intermediate form, and 12, 13, or 22 a form resembling the mature protein [44]. Our observations, with those of Tsutsumi et al. [44], suggest that removing variable portions of N-terminus is still sufficient for cytokine activation. Additional factors may regulate IL-18 activation beyond the primary sequence in this region, such as stabilizing reactions in the secondary structure burying side chains recognized by LasB or other promiscuous proteases in the environment.

We further observed that primary keratinocytes secrete a more diverse set of cytokines relative to immortalized cell lines (**Fig 1**). One of them is IL-18, a cytokine that serves as a crucial bridge from the innate immune responses to the adaptive immune cells. However, all cytokine profiles were variable between cell lines, which are commonly used or interpreted interchangeably. Therefore, caution should be exercised when immortalized cells are used for modeling host-pathogen interactions, and autocrine or paracrine effects of these cytokines should be considered when there is potential to impact experimental findings. Furthermore, antimicrobial effectors are commonly co-regulated by many of the same signaling transduction networks, and these potential defects in each line can mask pathogen phenotypes. Furthermore, unlike keratinocytes, endothelial cells did not release IL-18. Therefore, extracellular activation of IL-18 by SpeB may be most important within the skin, giving an implicit role in priming early innate and adaptive immune responses to infection.

## Materials and methods

### Ethics statement

This study was conducted according to the principles expressed in the Declaration of Helsinki. Blood was collected from healthy adult volunteers under informed consent under approval number IRB00110971 granted by the Institutional Review Board of Emory University. Approval for animal experiments was granted by the Institutional Animal Care and Use Committees of Emory University.

### Bacterial strains

GAS strain 5448, representative of the pandemic M1T1 clone, and its isogenic *ΔspeB*, *Δemm1*, *Δslo*, *ΔspyA*, *ΔcepA*, *Δmac*, *ΔcovRS*, JRS4, M4C20, *Pseudomonas aeruginosa* PA01, *Staphylococcus aureus* USA 300, and *Lactococcus lactis* carrying pSpeB have been previously described [36,43,62,66]. HKU16, a M12 scarlet fever isolate, was provided by Mark Walker and previously described [67]. *Streptococcus anginosus* F0211, *Streptococcus intermedius* F0413, and *Streptococcus mitis* F0392 were obtained through BEI Resources, NIAID, NIH. *Streptococcus constellatus*, *Staphylococcus hominus*, *Staphylococcus hominus*, and additional GAS clones are all clinical isolates provided through the Emory University Investigational Clinical Microbiology Core. All bacteria were statically grown overnight at 37°C with 5% CO_2_ in Todd Hewitt broth (THB, Difco), washed two times with phosphate-buffered saline (PBS), and diluted to a multiplicity of infection (MOI) of 10 for *in vitro* infections. Selection for bacteria carrying pSpeB was maintained with spectinomycin (Sigma) 100 μg/ml and expression was controlled with titrations of anhydrotetracycline (Cayman Chemical) as previously [66].

### Cell culture

A-431 lung epidermal cells (ATCC), Detroit 562 pharyngeal cells (ATCC), HaCat cells (unavailable from a repository, provided by C. Quave, Emory), and HEp-2 laryngeal cells (ATCC) were cultured in Eagle’s Minimum Essential Medium (Gibco) supplemented with 10% heat-inactivated fetal bovine serum (hiFBS, Atlanta Biologicals). HEK-Blue IL-18 Reporter cells (Invivogen) were cultured in Dulbecco’s Modified Eagle’s Medium (Gibco) with 10% hiFBS. 100 U/mL penicillin, 100 μg/mL streptomycin, and 100 μg/mL normocin (all Invivogen) were supplemented during routine culture and omitted during experimental infections, unless otherwise noted.

### Primary cell culture

Primary human keratinocytes were supplied by Lonza and cultured in Growth Medium 2 with supplement (C-20011, C-39011; PromoCell). Primary umbilical vein endothelial cells were supplied by Lifeline Cell Technology and cultured in endothelial cell growth medium (C-22010; PromoCell). Mouse primary keratinocytes were isolated from the tails of C57Bl/6 mice and cultured as previously [42]. Peripheral blood mononuclear cells (PBMCs) were isolated from donor blood by Ficol Histopaque 1077, then frozen at 5x10^6^/mL in FBS/DMSO until use. Cells were thawed and cultured in RPMI + 10% hiFBS. T lymphocytes were depleted from PBMCs using positive selection for CD2 by MACS standard protocol (Militenyi). All cells were maintained at 37°C and 5% CO_2_. For PBMC-NHEK coculture, NEHKs were plated at 4x10^4^/mL in keratinocyte growth media in TC-treated 96 well plates and grown for 48 h. The media was then aspirated and replaced with 100uL 1x10^6^/mL PBMCs in RPMI + 10% FBS. For cell lysis, 0.05% triton X-100 (Sigma) was added 5 min. Other cell treatments are 5 μM caspase-1 inhibitor YVAD-fmk (R&D Systems), 50 μM SpeB inhibitor E-64 (Sigma), 20 μg/mL anti-IL-18 IgG (Abcam), or 10 μM caspase-1 inhibitor VX-765 (Invivogen), for the full duration of the experiment.

### Cell measurements

The relative levels of selected human cytokines and chemokines were determined in parallel using a membrane-based antibody array (ARY005B, R&D Systems). Cells were seeded at 7.5 x 10^5^ cells per well in a tissue-culture treated six-well plate. Cells were infected at an MOI of 10, and bacteria spun onto cells at 160 x g for three minutes. 1 mL of cell supernatant was collected after six hours of infection, incubated on the membrane overnight at 4°C and developed following the manufacturer’s protocol. Chemiluminescence was measured using the ChemiDoc MP imaging system, and mean pixel density was quantified using ImageJ. Only proinflammatory cytokines made by at least one of the cells lines under study are presented graphically. IL-18 signaling activity was measured in 50 μL volumes of cell supernatant using HEK-Blue IL-18 Reporter cells (Invivogen) and secreted alkaline phosphatase activity measured after 18 h incubation as previously described [68]. For murine cells, supernatants were concentrated 4x due to the lower sensitivity of detection of mouse IL-18. Both were normalized to known quantities of recombinant mature IL-18 (Invivogen). Total IL-18, IL-1β, and IFN-γ were quantified by ELISA following the dilutions, standards, and protocol of the manufacturer (R&D Systems). Cell lysis was measured by quantifying released LDH, compared to untreated and Triton X-100 treated controls, following the manufacturer’s protocol (CytoTox 96; Promega). Cell lysis and caspase-1 activation was determined by propidium iodide and Fam-YVAD-FMK staining by the manufacturer’s protocol (Immunochemistry Technologies). Immunofluorescence and brightfield images were collected using an AxioObserver D1 microscope (Zeiss) and exported with Zen Pro 2 (Zeiss). All images were acquired with the same acquisition settings, and brightness, contrast, and all other parameters were identical between samples.

### Protein purification

The fully spliced coding sequence for human *il18* and murine *il18*, encoding pro-IL-18 for each species, was generated by gene synthesis for expression from pETPP [36] to generate pETPP-hIL18 and pETPP-mIL18. Expression was induced from the plasmids in BL21 cell induced with 0.2 mM IPTG (Sigma) overnight at 18°C. Bacterial cell pellets were suspended in 10 mL of phosphate-buffered saline (PBS, pH 7.4). Cells were completely lysed by sonication at 40% amplitude for two minutes for 30 seconds at 10 second intervals and centrifugation at 7500 x g for 10 minutes. Lysate was through Talon gravity columns loaded with HisPur Cobalt Resin (Thermo Scientific), washed with PBS, and eluted in PBS supplemented with 300 mM imidazole. SpeB was purified as previously [43] and found to be >99% pure by SDS-PAGE. The specific activity of SpeB was measured by incubation with the specific FRET peptide Mca-IFFDTWDNE-Lys-Dnp (CPC Scientific) in PBS with 1 mM DTT and measuring the change in fluorescence, as previously [43]. Cleavage of purified human and murine pro-IL-18 was performed as previously with pro-IL-1β [43], with SpeB (100 ng) and Caspase-1 (1U, Novus) in PBS with 2 mM dithiothreitol 2 h at 37°C. Reactions were stopped by the addition of Laemmli buffer, 10% β-ME, and 1 x SDS loading buffer solution, then boiled at 95°C for 5 min. Samples were analyzed by SDS-PAGE on Tris-Glycine gels (Invitrogen) and visualized with AquaStain (Bulldog Bio). Samples for Edman Degradation were wet transferred to PVDF membrane and visualized with SimplyBlue Safe Stain (Invitrogen), then sequenced on an ABI 494 Protein Sequencer by the Tufts University Core Facility.

### Mouse protocols

GAS cultures were grown statically overnight at 37°C in Todd-Hewitt broth, washed 2x in PBS, and 1x10^8^ colony-forming units (CFU) in 100 μL of PBS injected subcutaneously into seven-week-old C57BL/6 or *il18*^-/-^ male or female mice (Jackson Laboratories) as previously [43]. After 72 h, mice were euthanized by CO_2_ asphyxiation, lesions removed, homogenized, and CFU enumeration by dilution plating on blood agar plates.

### Statistics and data analysis

Values are expressed as mean ± standard deviation. Differences between groups were analyzed using a 1-way analysis of variance with Dunnett multiple comparisons analysis unless otherwise indicated. Differences are considered statistically significant at a P value of <0.05 using GraphPad Prism v9 software. Principal Component Analysis (PCA) was used to reduce the number of variables needed to adequately describe differences in cytokine profiles; each condition was treated as an independent variable, and multivariate analysis was performed in Prism v9. Protein models were visualized using PyMol 2.3.3. Diagrams created with BioRender.com.

## Supporting information

S1 Fig Images of cytokine arrays for quantification of secreted cytokines. (PDF)
