# Supplementary material for: Constitutive secretion of pro-IL-18 allows keratinocytes to initiate inflammation during bacterial infection
Source: PLoS Pathog. 2023 Apr 17;19(4):e1011321. doi: 10.1371/journal.ppat.1011321 (PMC10138833; doi:10.1371/journal.ppat.1011321)
Supplement: S1 Fig — (PDF) [file ppat.1011321.s001.pdf]

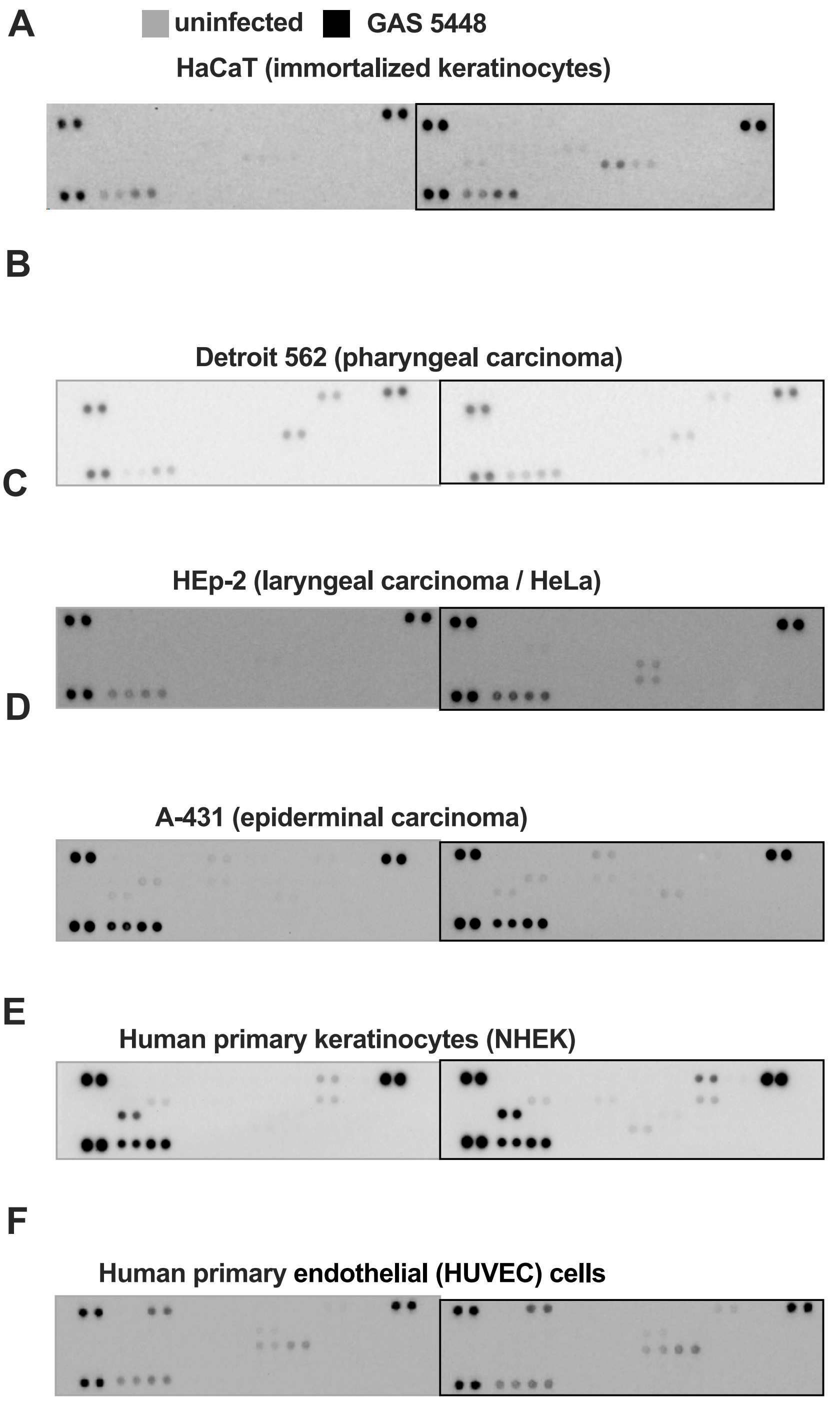

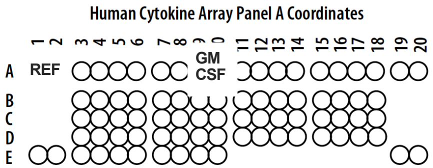

APPENDIX

Refer to the table below for the Human Cytokine Array coordinates.

Coordinate	Target/Control	Entrez Gene ID	Alternate Nomenclature
A1, A2	Reference Spots	N/A	———
A3, A4	CCL1/I-309	6346	P500, SCYA1, SCYA2, TCA-3
A5, A6	CCL2/MCP-1	6347	MCAF
A7, A8	MIP-1α/MIP-1β	6348/6351	CCL3/CCL4
A9, A10	CCL5/RANTES	6352	———
A11, A12	CD40 Ligand/TNFSF5	959	CD154, CD40LG, gp39, TRAP
A13, A14	Complement Component C5/C5a	727	C5/C5a
A15, A16	CXCL1/GROα	2919	CINC-1, KC
A17, A18	CXCL10/IP-10	3627	CRG-2
A19, A20	Reference Spots	N/A	———
B3, B4	CXCL11/I-TAC	6373	β-R1, H174
B5, B6	CXCL12/SDF-1	6387	PBSF
B7, B8	G-CSF	1440	CSFβ, CSF-3
B9, B10	GM-CSF	1437	CSFα, CSF-2
B11, B12	ICAM-1/CD54	3383	———
B13, B14	IFN-γ	3458	Type II IFN
B15, B16	IL-1α/IL-1F1	3552	———
B17, B18	IL-1β/IL-1F2	3553	———
C3, C4	IL-1ra/IL-1F3	3557	———
C5, C6	IL-2	3558	TCGF
C7, C8	IL-4	3565	BCDF, BSF1
C9, C10	IL-5	3567	———
C11, C12	IL-6	3569	BSF-2
C13, C14	IL-8	3576	CXCL8, GCP1, NAP1
C15, C16	IL-10	3586	CSIF
C17, C18	IL-12 p70	3592/3593	CLMF p35

APPENDIX *CONTINUED*

Coordinate	Target/Control	Entrez Gene ID	Alternate Nomenclature
D3, D4	IL-13	3596	———
D5, D6	IL-16	3603	LCF
D7, D8	IL-17A	3605	CTLA-8
D9, D10	IL-17E	64806	IL-25
D11, D12	IL-18/IL-1F4	3606	IGIF
D13, D14	IL-21	59067	———
D15, D16	IL-27	246778	IL-27 A
D17, D18	IL-32α	9235	
E1, E2	Reference Spots	N/A	———
E3, E4	MIF	4282	GIF, DER6
E5, E6	Serpin E1/PAI-1	5054	Nexin, PLANH1
E7, E8	TNF-α	7124	TNFSF1A
E9, E10	TREM-1	54210	CD354
E19, E20	Negative Control	N/A	———

Detected in no sample: CCL1, MIP, CCL5, CXCL11, G-CSF, IFN-γ, IL-1β, IL-2, IL-4, IL-5, IL-10, IL-12, IL-13, IL-16, IL-21, IL-27, IL-32α, TNFα
